# Effect of Microbial Short-Chain Fatty Acids on CYP3A4-Mediated Metabolic Activation of Human Pluripotent Stem Cell-Derived Liver Organoids

**DOI:** 10.3390/cells10010126

**Published:** 2021-01-11

**Authors:** Seon Ju Mun, Jaeseo Lee, Kyung-Sook Chung, Mi-Young Son, Myung Jin Son

**Affiliations:** 1 Stem Cell Convergence Research Center, Korea Research Institute of Bioscience and Biotechnology (KRIBB), 125 Gwahak-ro, Yuseong-gu, Daejeon 34141, Korea; sjmoon@kribb.re.kr (S.J.M.); lljaeseo@kribb.re.kr (J.L.); kschung@kribb.re.kr (K.-S.C.); 2 Department of Functional Genomics, Korea University of Science & Technology (UST), 217 Gajungro, Yuseong-gu, Daejeon 34113, Korea; 3 Biomedical Translational Research Center, KRIBB, 125 Gwahak-ro, Yuseong-gu, Daejeon 34141, Korea

**Keywords:** liver organoids, hepatotoxicity, induced pluripotent stem cells, short-chain fatty acids

## Abstract

The early and accurate prediction of the hepatotoxicity of new drug targets during nonclinical drug development is important to avoid postmarketing drug withdrawals and late-stage failures. We previously established long-term expandable and functional human-induced pluripotent stem cell (iPSC)-derived liver organoids as an alternative source for primary human hepatocytes. However, PSC-derived organoids are known to present immature fetal characteristics. Here, we treated these liver organoids with microbial short-chain fatty acids (SCFAs) to improve metabolic maturation based on microenvironmental changes in the liver during postnatal development. The effects of the three main SCFA components (acetate, propionate, and butyrate) and their mixture on liver organoids were determined. Propionate (1 µM) significantly promoted the *CYP3A4*/*CYP3A7* expression ratio, and acetate (1 µM), propionate (1 µM), and butyrate (1 µM) combination treatment, compared to no treatment (control), substantially increased CYP3A4 activity and albumin secretion, as well as gene expression. More importantly, mixed SCFA treatment accurately revealed troglitazone-induced hepatotoxicity, which was redeemed on a potent CYP3A4 inhibitor ketoconazole treatment. Overall, we determined, for the first time, that SCFA mixture treatment might contribute to the accurate evaluation of the CYP3A4-dependent drug toxicity by improving metabolic activation, including CYP3A4 expression, of liver organoids.

## 1. Introduction

Drug-induced liver injury (DILI) is the main cause of postmarketing drug withdrawals, and early and accurate hepatotoxicity prediction of drugs is critical in nonclinical drug development [1,2]. Primary human hepatocytes (PHHs) are mainly used in toxicity assessment due to their functionality. However, because of their low availability and viability, alternative cell sources are required [3]. Stem cells are a useful source of human liver cells. More specifically, induced pluripotent stem cells (iPSCs) are a valuable source, capable of indefinitely providing patient-specific parenchymal and nonparenchymal human liver cells [4]. Various technologies have been developed, from the differentiation of hepatocytes in conventional two-dimensional (2D) cultures [5,6] to the generation of hepatic organoids, a more advanced three-dimensional (3D) liver model, from PSCs [7,8]. Recent advances in induced pluripotent stem cells (iPSC) and organoid technology have provided patient-specific and renewable cell sources. However, iPSC-derived organoids reportedly present immature fetal characteristics in vitro [9,10,11].

In previous studies, we established expandable and functional PSC-derived human hepatic organoids, which showed mature hepatic functions, such as serum protein production and drug detoxification activity, and were as functionally competent as adult tissue-derived liver organoids [12,13]. Continuous expansion for >90 passages without growth retardation and long-term cryopreservation is currently possible. Cytochrome p450 (*CYP*) enzyme expression and CYP3A4 activity were incomparably higher in the organoids than in 2D differentiated hepatocytes. However, they did not reach the levels in PHHs or adult liver tissue, as previously described [14,15], as the liver similarity score quantified by an RNA-sequencing-based validation algorithm [16] remained at 60.22% [12]. Therefore, we considered various strategies to improve the hepatic maturation of liver organoids.

The liver of a newborn human infant is functionally immature, and anatomical and functional liver maturation is achieved during the first week of life [14]. Dynamic environmental changes, such as those in oxygen circulation and enteral nutrition, which contribute to liver microenvironmental changes through the periportal blood supply, occur after birth. CYP maturation in the liver is primarily important for metabolic activity and xenobiotic detoxification. Moreover, CYP expression level is only ~30% at birth and then gradually increases to the adult level during the first year of life [17]. Expression of CYP3A7, a fetal counterpart of CYP3A4, decreases after birth, while that of CYP3A4 gradually increases with albumin (ALB) expression [18]. Full functional maturation, including drug metabolism, bile acid synthesis, and amino acid transport, takes as long as two years [17,19]. Postpartum changes in the liver microenvironment, such as those in enteral nutrition and oxygen circulation, have been suggested as the main cues for the final maturation of hepatic cells [20].

Importantly, the effects of the intestinal microbiome on the liver via the gut–liver axis showed that germ-free mice presented different expression patterns of CYP enzymes compared with wild-type mice [21]. Notably, the important roles of gut microbiota-derived metabolites, such as secondary bile acids and short-chain fatty acids (SCFAs), in regulating liver functions under physiological and pathological conditions have emerged [22]. SCFAs, such as acetate, propionate, and butyrate, are produced by gut microbe-mediated fermentation of dietary carbohydrates, which are primarily used as an energy source for the gut epithelium, and enter the liver through the portal vein [23]. SCFAs contribute to the main liver functions, including lipid metabolism and gluconeogenesis [24], resulting in controversial promotion [25,26] or prevention [27,28] of liver disease based on the SCFA subtypes and levels. However, the effects of SCFAs on hepatic maturation are not well known. Therefore, we determined the effects of the three main SCFA components (acetate, propionate, and butyrate) on the hepatic maturation of liver organoids.

## 2. Materials and Methods

### 2.1. Reagents

Sodium acetate, sodium propionate, sodium butyrate, lithocholic acid, and ketoconazole were purchased from Sigma-Aldrich (St. Louis, MO, USA). Troglitazone was purchased from Toronto Research Chemicals (North York, ON, Canada).

### 2.2. Organoid Culture

Liver organoids were generated from two human iPSC lines. One was reprogrammed from human foreskin fibroblasts (CRL-2097; American Type Culture Collection (ATCC), Manassas, VA, USA) using the Sendai virus, as previously described [12], and the other was normal human bone marrow blood cell-derived CMC-hiPSC-003, provided by the National Stem Cell Bank of Korea (Korea National Institute of Health), originally provided by the Catholic University. The iPSCs were differentiated stepwise into definitive endoderm, hepatic endoderm, and hepatocytes following the liver developmental process [13]. Generated 3D cyst-shaped organoids were embedded in Matrigel™ (Corning, Corning, NY, USA) and expanded in hepatic medium (HM) [12]. The organoids were routinely passaged every week using a surgical blade and re-embedded at a ratio of 1:3–1:5 in fresh Matrigel. For further hepatic differentiation, HM was replaced with expansion medium (EM) [12] supplemented with BMP7 (20 ng/mL; PeproTech, Cranbury, NJ, USA) the day after seeding. EM was then replaced with differentiation medium (DM) [12] after 3 days and then replaced once every 2 days.

### 2.3. Quantitative Real-Time Polymerase Chain Reaction (qPCR)

Total RNA was extracted using the TRIzol reagent (Thermo Fisher Scientific, Waltham, MA, USA), and cDNA was synthesized using a TOP ScriptTM RT DryMIX, dT18 plus (Ezynomics, Daejeon, Korea) following the manufacturer’s instructions. qPCR was performed using a specific primer for each gene (Table S1) and Fast SYBR^®^ Green Master Mix (Applied Biosystems, Waltham, MA, USA) on a 7500 Fast Real-Time PCR System (Applied Biosystems).

### 2.4. Immunocytochemistry

Organoids were fixed with 4% paraformaldehyde (PFA; Biosesang, Seongnam-si, Gyeonggi-do, Korea) in phosphate-buffered saline (PBS; Thermo Fisher Scientific) and permeabilized with 0.25% Triton X-100 (Sigma-Aldrich) at room temperature (RT) for each 15 min. The organoids were blocked with 4% bovine serum albumin (Sigma-Aldrich) at RT for 1 h and incubated overnight with each primary antibody (Table S2) at 4 °C. The organoids were washed with 0.05% Tween-20 (Sigma-Aldrich)/PBS and then stained with Alexa Fluor^®^-conjugated secondary antibodies (Thermo Fisher Scientific) at RT for 1 h. The nuclei were counterstained with 4′,6-diamidino-2-phenylindole (DAPI) (Sigma-Aldrich), and the fluorescence images were observed under a Zeiss confocal microscope (Oberkochen, Germany).

### 2.5. Periodic Acid–Schiff (PAS) Staining and Indocyanine Green (ICG) Uptake

To determine glycogen storage, organoids were fixed with 4% PFA at RT for 15 min and merged overnight in 30% sucrose (Sigma-Aldrich) at 4 °C. The samples were frozen in an optimal-cutting-temperature compound (Sakura Finetek, Torrance, CA, USA) and cut into 10 μm thick slices using a cryostat microtome (Leica, Wetzlar, Germany) at −20 °C. Frozen sections were stained with PAS (IHC World, Ellicott City, MD, USA), according to the manufacturer’s protocol. For ICG uptake, Matrigel-embedded organoids were washed with cold PBS and then incubated with ICG (1 mg/mL; Sigma-Aldrich) in a 5% CO2 incubator at 37 °C for 15 min. Images of PAS staining and ICG uptake were observed under an Olympus microscope (Shinjuku-ku, Tokyo, Japan).

### 2.6. ALB Secretion Assay

The cultured medium was collected 24 h after replacement with fresh medium, and ALB secretion was measured using an enzyme-linked immunosorbent assay kit (Bethyl Laboratories, Inc, Montgomery, AL, USA) according to manufacturer instructions. Absorbance was detected using a Spectra Max M3 microplate reader (Molecular Devices, Sunnyvale, CA, USA), and data were normalized to total cell number counted by Trypan Blue exclusion.

### 2.7. CYP3A4 Activity Assay

CYP3A4 activity was analyzed using a P450-Glo assay kit (V9002; Promega, Madison, WI, USA) according to manufacturer instructions. Luciferase activity was measured using a luminometer (GloMax Navigator; Promega), and the results were normalized to a total cell number counted by Trypan Blue exclusion.

### 2.8. Flow Cytometry Analysis

Liver organoids were dissociated into single cells with TrypLE (Thermo Fisher Scientific) for 10 min at 37 °C and then filtered using 30 μm mesh (BD Biosciences, Franklin Lakes, NJ, USA). The single cells were fixed in 4% paraformaldehyde (Biosesang) for 15 min at RT and then permeabilized with 0.25% Triton X-100 (Sigma-Aldrich) for 15 min at RT. The cells were incubated with the primary antibody in 0.5% bovine serum albumin (Sigma-Aldrich) and 2 mM EDTA (Thermo Fisher Scientific) in PBS for 30 min at 4 °C, followed by washing with the buffer, and then incubation with Alexa Fluor-conjugated secondary antibody (Thermo Fisher Scientific) for 30 min at 4 °C and analysis using the BD AccuriTM C6 system (BD Biosciences). The list of antibodies used in this study is presented in Supplementary Table S2. For live/dead cell counting, dissociated single cells were stained with Trypan Blue and counted using a Countess II automated cell counter (AMQAX1000; Thermo Fisher Scientific).

### 2.9. Drug Toxicity Analysis

Cryopreserved PHHs (454543; Corning) were thawed with cryopreserved hepatocyte plating medium and cryopreserved hepatocyte recovery medium (Thermo Fisher Scientific) according to manufacturer instructions in a Matrigel-coated dish at 2 × 10^5^ cells/cm^2^. The medium was replaced with fresh hepatocyte culture medium (cc-3199; Lonza Group AG, Basel, Switzerland) at 4 to 6 h after thawing. HepG2 cells (ATCC) were cultured in Dulbecco Modified Eagle Medium (Thermo Fisher Scientific) supplemented with 10% fetal bovine serum (Corning) and 100 U/mL penicillin–streptomycin (Thermo Fisher Scientific). To evaluate CYP3A4-mediated hepatotoxicity, DM-differentiated organoids were treated with troglitazone (50 μM) and propionate (1 μM), an SCFA mixture (acetate:propionate:butyrate = 1:1:1), or CYP3A4 inhibitor ketoconazole (1 μM) 3 days after differentiation for another 6 days. PHHs and HepG2 cells were seeded in 96-well plates, and the following day, a troglitazone and SCFA mixture was treated daily for 3 days. Toxicity was evaluated using EZ-cytox (Dogenbio, Seoul, Korea) following the manufacturer’s instructions. The absorbance of the medium was measured using a Spectra Max M3 microplate reader (Molecular Devices, San Jose, CA, USA).

### 2.10. Statistical Analysis

All data were obtained from more than three independent biological replicates. The graphs represent the mean ± standard error of the mean (SEM) of triplicate samples. Student’s *t*-test was used to evaluate intergroup comparisons, and *p* < 0.05 indicated statistical significance.

## 3. Results

### 3.1. Generation of Functional Liver Organoids from Human iPSCs

**Figure 1:**
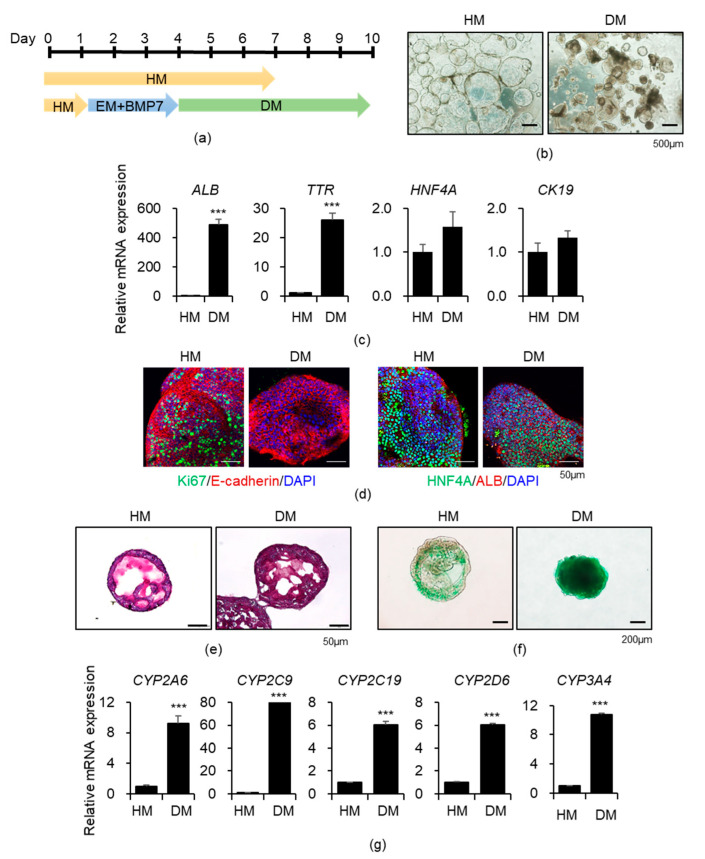
Characterization of functional liver organoids generated from human induced pluripotent stem cells (hiPSCs). (**a**) Scheme of liver organoid differentiation is shown. HM, hepatic medium; EM, expansion medium; DM, differentiation medium. (**b**) Representative morphology of HM-maintained proliferating organoids (left) and DM-differentiated organoids (right). (**c**) *ALB*, *TTR*, *HNF4A*, and *CK19* mRNA expression levels in the HM and DM organoids. (**d**) Representative immunofluorescence images of HM- and DM-cultured organoids stained with each indicated antibody. (**e**) Representative images of accumulated glycogen stained with periodic acid–Schiff (PAS) in HM- and DM-cultured organoids. (**f**) Representative images of indocyanine green (ICG) uptake by HM- and DM-cultured organoids. (**g**) *CYPs* mRNA expression levels in HM- and DM-cultured organoids. Data are presented as the mean ± SEM (*n* = 3) and analyzed by Student’s *t*-test. *** *p* < 0.001.

We previously established long-term expandable and functional human iPSC-derived liver organoids [12,13]. HM-maintained proliferating organoids were further differentiated in the DM (Figure 1a,b). Upon differentiation, expression levels of mature hepatocyte markers *ALB* and *transthyretin* (*TTR)* in the DM were robustly increased, whereas those of the early hepatocyte marker *hepatocyte nuclear factor 4α* (*HNF4A)* and the biliary/progenitor cell marker *CK19* in the DM were similar to those in the HM (Figure 1c). Additionally, high protein expression of Ki67-positive proliferating cells was detected in the HM, and expression of the epithelial marker E-cadherin and the hepatocyte markers HNF4A and ALB was clearly observed in both the HM and DM via immunostaining (Figure 1d). Glycogen storage was detected by PAS staining (Figure 1e) and ICG uptake (Figure 1f), which represent mature hepatic functioning, and was increased in the DM. Moreover, the expression levels of the drug-metabolizing CYP enzymes were substantially increased in the DM (Figure 1g). Next, we examined the effects of SCFAs on the liver organoids, especially on their drug metabolizing activity.

### 3.2. Effects of Acetate, Propionate, and Butyrate on iPSC-Derived Liver Organoids

**Figure 2:**
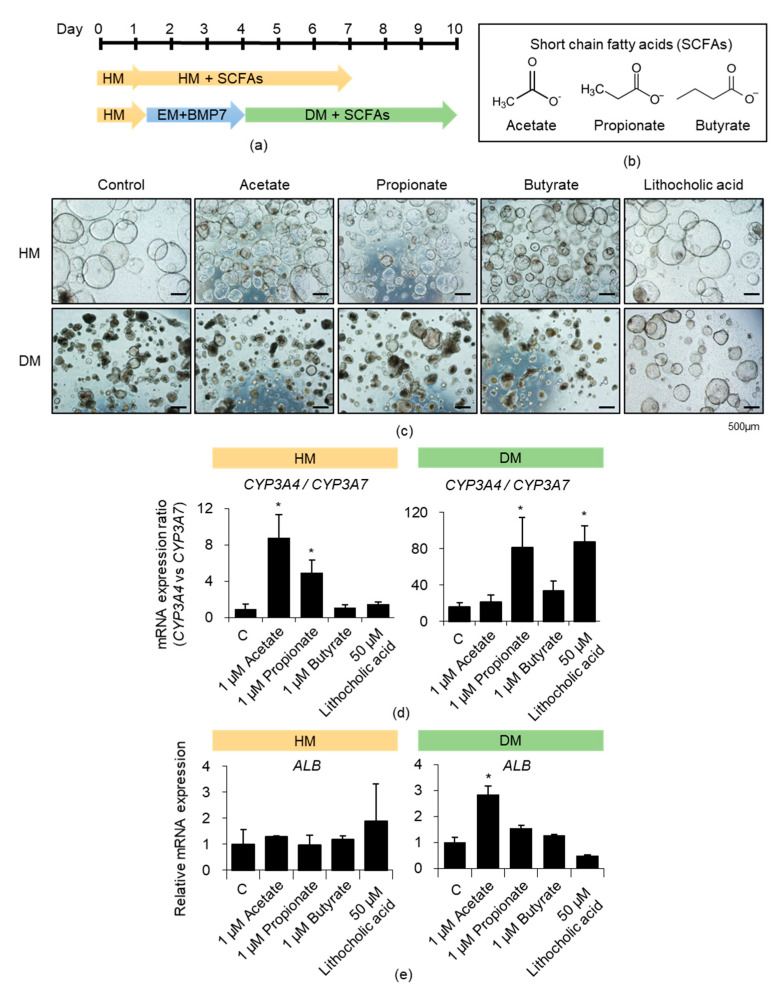
Effects of acetate, propionate, and butyrate on iPSC-derived liver organoids. (**a**) Scheme of short-chain fatty acids (SCFAs) treatment is shown. (**b**) Chemical structures of the SCFAs. (**c**) Representative morphology of SCFA- or lithocholic acid-treated HM (upper)- and DM (lower)-cultured organoids. (**d**) The *CYP3A4*/*CYP3A7* mRNA expression ratios in the HM (left)- and DM (right)-cultured organoids under each indicated condition. (**e**) *ALB* mRNA expression levels in the HM (left)- and DM (right)-cultured organoids under each indicated condition. Data are presented as the mean ± SEM (*n* = 3) and analyzed by Student’s *t*-test. * *p* < 0.05.

We treated the liver organoids in both the HM and DM with the three main SCFA components: acetate, propionate, and butyrate (Figure 2a,b). The mRNA expression levels of *CYP3A4* and its fetal counterpart *CYP3A7* were determined after treatment with SCFAs of various concentrations based on their native contents [29] (Figure S1). Acetate (0.5 and 5 μM) and propionate (1 μM) increased *CYP3A4* expression and decreased *CYP3A7* expression in the HM (Figure S1a). Propionate (1 μM) showed similar effects in the DM (Figure S1b). Therefore, we compared the effects of SCFAs (1 μM) and the positive control lithocholic acid (50 μM), which has been previously reported to increase pregnane X receptor (PXR) and CYP3A4 expression in fetal hepatocytes [15] (Figure 2c). Acetate and propionate significantly promoted the *CYP3A4*/*CYP3A7* expression ratio in the HM, and propionate substantially increased the ratio to a level similar to that obtained on lithocholic acid treatment in the DM (Figure 2d). Acetate also increased *ALB* expression level in the DM (Figure 2e). SCFAs additionally enhanced *CYP2A6*, *CYP2C9*, and *CYP2C19* expression levels in the DM (Figure S2). Therefore, these data indicated that SCFAs might partially improve the metabolic maturation of liver organoids. Thus, we then determined the effects of the SCFA mixture.

### 3.3. Effects of SCFA Mixture on iPSC-Derived Liver Organoids

**Figure 3:**
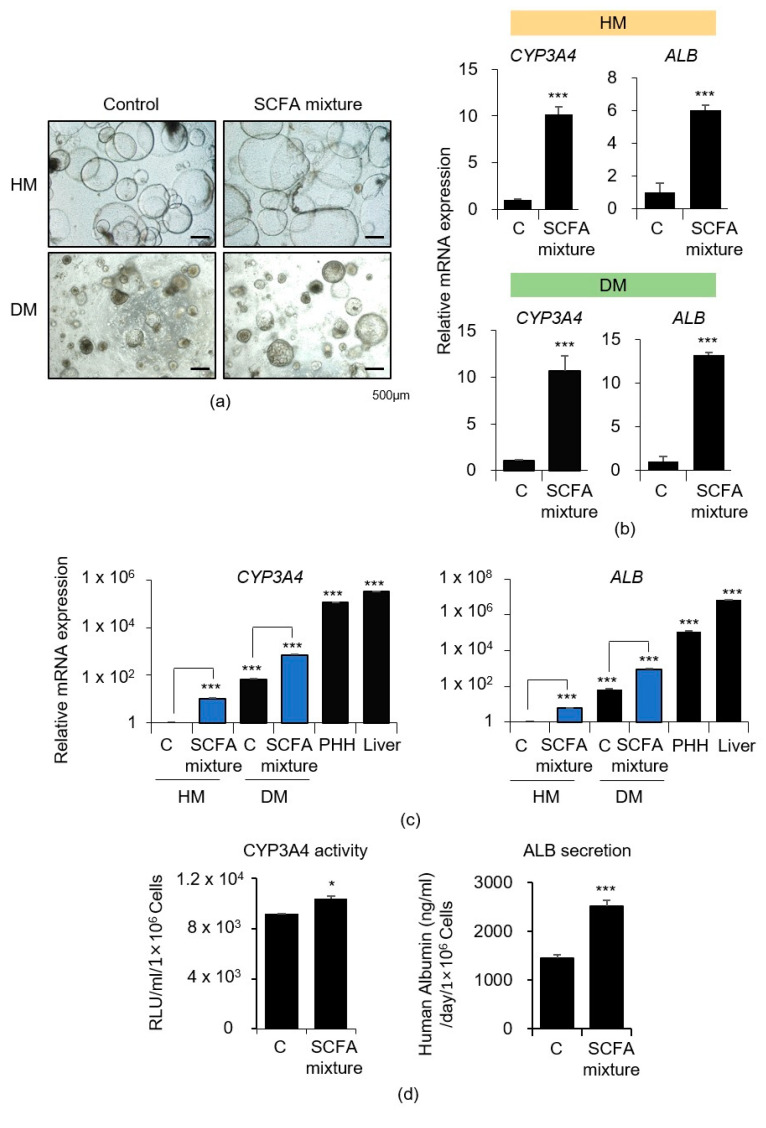
Effects of SCFA mixture on iPSCs-derived liver organoids. (**a**) Representative morphology of the untreated control and SCFA mixture-treated HM (upper)- and DM (lower)-cultured organoids are shown. (**b**) *CYP3A4* and *ALB* mRNA expression levels in the control and SCFA mixture-treated HM (upper)- and DM (lower)-cultured organoids are shown. (**c**) *CYP3A4* (left) and *ALB* (right) mRNA expression levels in the untreated control, SCFA mixture-treated group, primary human hepatocytes (PHHs), and adult liver tissue are shown. (**d**) CYP3A4 activity (left) and ALB secretion (right) in control and SCFA mixture-treated DM organoids are shown. Data are presented as the mean ± SEM (*n* = 3) and analyzed by Student’s *t*-test. * *p* < 0.05 and *** *p* < 0.001.

We treated the liver organoids in both the HM and DM with a combination of acetate (1 μM), propionate (1 μM), and butyrate (1 μM) for six days (Figure 3a). *CYP3A4* and *ALB* mRNA expression levels in the HM and DM were potently increased on mixed SCFA treatment (Figure 3b). These levels were still lower than those obtained in PHHs or adult liver tissue. However, SCFA combination treatment, compared to no treatment (control), significantly upregulated *CYP3A4* and *ALB* expression levels (Figure 3c). This effect was reproducibly observed in another human iPSC line (Figure S3), as liver organoids from bone marrow blood cell-derived CMC-hiPSC-003 cells showed significantly elevated *CYP3A4* and *ALB* mRNA levels following treatment with the SCFA mixture in both HM and DM (Figure S3a,b)

Furthermore, SCFA mixture treatment significantly increased CYP3A4 enzyme activity and ALB secretion (Figure 3d), as well as gene expression. ALB-positive populations were increased from 56.1 to 89.7% according to flow cytometry analysis following treatment with the SCFA mixture (Figure S4a). As the dead-cell ratios did not differ significantly between the two groups (13.68 vs. 18.00%, respectively) (Figure S4b), it is possible that increased ALB expression following SCFA treatment may not be due to the selection of a specific cell population but is a consequence of the maturation of each cell.

### 3.4. Effects of SCFAs on CYP3A4-Mediated Drug Toxicity in iPSC-Derived Liver Organoids

**Figure 4:**
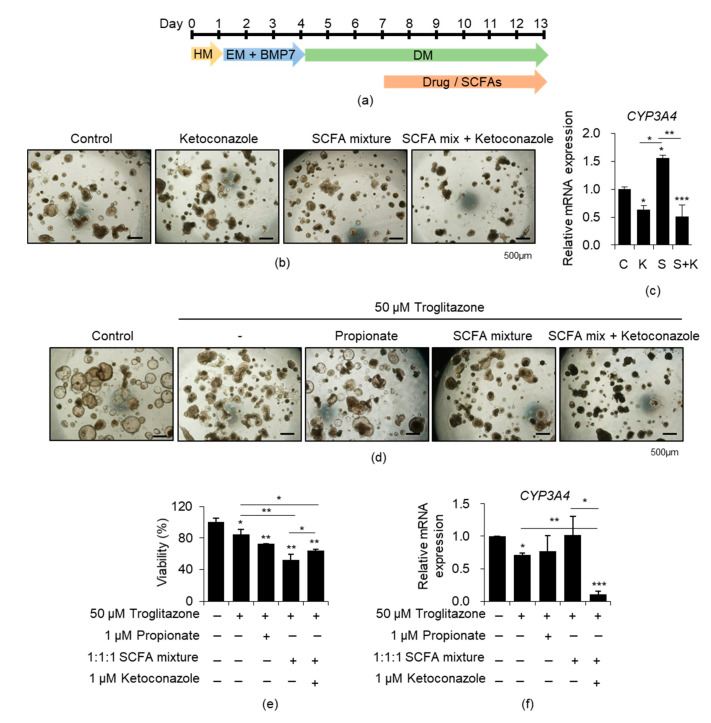
Effects of SCFAs on CYP3A4-mediated drug toxicity in iPSCs-derived liver organoids. (**a**) Scheme of drug and SCFAs treatment. (**b**) Representative morphology of DM-cultured organoids under each indicated treatment condition. (**c**) *CYP3A4* mRNA expression levels under each indicated condition. (**d**) Representative morphology of the untreated control and the troglitazone (hepatotoxic drug)-treated group under each indicated condition. (**e**) Relative cell viability under each indicated condition. (**f**) *CYP3A4* mRNA expression levels under each indicated condition. Data are presented as the mean ± SEM (*n* = 3) and analyzed by Student’s *t*-test. * *p* < 0.05, ** *p* < 0.01, and *** *p* < 0.001.

Next, we examined the effects of SCFAs on the drug toxicity response of liver organoids based on the increase in the CYP3A4 expression (Figure 4). The toxicity test was performed in DM condition, which led to more expression level of *CYP3A4* compared with that in HM (Figure 1g). Drug treatment was started three days after differentiation to consider the possibility of a differentiation defect due to drug toxicity (Figure 4a). *CYP3A4* expression level three days after differentiation was sufficiently similar to that after six days (Figure S3). Thus, we treated the organoids with the drug and SCFAs three days after differentiation for another six days (Figure 4a). Troglitazone is an antidiabetic drug that was withdrawn from the market due to serious hepatotoxicity, and the possibility of drug-induced idiosyncratic hepatotoxicity by CYP3A4-mediated reactive metabolites was suggested [30,31]. Therefore, we first examined the effects of a potent CYP3A4 inhibitor, ketoconazole, to confirm the CYP3A4-dependent activity (Figure 4b). Ketoconazole strongly inhibited the mixed SCFA treatment-mediated increase in *CYP3A4* expression (Figure 4c). Finally, we determined the effects of propionate and the SCFA mixture on troglitazone-induced hepatotoxicity (Figure 4d). Propionate and SCFA mixture treatments, compared with only troglitazone treatment, further decreased cell viability to 8.24 ± 0.31% and 34.09 ± 9.36%, respectively (Figure 4e). SCFA mixture treatment increased *CYP3A4* expression level (Figure 4f). The decrease in viability after SCFA mixture treatment recovered slightly (Figure 4e), and the *CYP3A4* expression level was strongly inhibited on treatment with ketoconazole (Figure 4f). However, cell viability was unaffected by treatment with propionate, the SCFA mixture, and ketoconazole, respectively (Figure S6a). Furthermore, the increased hepatotoxicity of troglitazone, along with increased *CYP3A4* expression following SCFA mixture treatment, was reproducibly observed in another human iPSC line (CMC-hiPSC-003) (Figure S6b–d).

In addition, we performed a drug toxicity test using PHHs and HepG2 cells (Figure S7). Significant increases in *CYP3A4* expression and decreased cell viability following SCFA mixture treatment with troglitazone were reproducibly observed in PHHs (Figure S7a–d). However, the effects of SCFAs or ketoconazole treatment were not detected in HepG2 cells, which showed barely detectable *CYP3A4* expression (Figure S7e–g). Overall, these results demonstrated that SCFA combination treatment might contribute to the accurate evaluation of CYP3A4-dependent toxicity of troglitazone by improving metabolic activation, including *CYP3A4* expression, of liver organoids.

## 4. Discussion

In human embryonic stem cell-derived hepatocytes and human fetal hepatoblasts, a secondary bile acid, lithocholic acid, produced by the intestinal microbiome, promotes the increase in CYP enzymes, such as CYP2C9 and CYP3A4 [15]. However, the functions of other microbiome-derived substances during liver metabolic maturation are largely unknown. In this study, we examined the effects of microbial SCFAs on the maturity enhancement of iPSC-derived liver organoids based on prenatal and postnatal liver microenvironmental changes [14]. The most abundant SCFAs in the gut lumen are acetate, propionate, and butyrate, found in a molar ratio of roughly 3:1:1 [29,32,33]. However, acetate exists at a lower percentage in the liver and is abundant in the systemic circulation, whereas butyrate and propionate are highly metabolized by the liver [29,34]. Butyrate, a histone deacetylase inhibitor, has been reported to promote direct hepatic differentiation [35] and CYP1A1 expression in colon epithelial cells [36] as an epigenetic modifier. Propionate increased hepatic lipid oxidation and inhibited hepatic lipid accumulation in a previous clinical study [37]. The regulatory effect of SCFAs on hepatic lipid accumulation and inflammation has also been determined, mainly in animal models [38,39]. However, a direct link between SCFAs and liver function, especially in relation to hepatic maturation, is limited in humans. In this regard, we evaluated the effects of SCFAs on hepatic maturation in vitro using our iPSC-derived liver organoid system.

Each type of SCFAs showed different patterns of functional enhancement of *CYP3A4*/*CYP3A7* and *ALB* expression levels, as shown in Figure 2d,e. However, SCFA mixture treatment dramatically increased both *CYP3A4* and *ALB* expression. *CYP3A4* and *ALB* mRNA expression levels were 10-fold and 13-fold higher, respectively, in DM-cultured liver organoids (in a further maturated state than HM-cultured liver organoids) after SCFA mixture treatment (Figure 3b). As a result, *CYP3A4* and *ALB* expression levels were 714-fold and 863-fold higher, respectively, in SCFA mixture-treated DM organoids than in untreated HM organoids, indicating a great improvement in the hepatic maturation of the liver organoids (Figure 3c). These effects were reproducibly examined in two sets of liver organoids derived from different individuals (Figure 3 and Figure S3). Moreover, CYP3A4 enzyme activity (Figure 3d) and ALB protein expression (Figure S4a) and secretion (Figure 3d) were also significantly improved by SCFA mixture treatment. To distinguish whether the effect of SCFAs on increased ALB levels was the result of cell maturation or selection, we quantified live cells and ALB-expressing cells following SCFA mixture treatment (Figure S4). The total number of cells was similar between control and treatment groups, and the staining intensity of ALB-positive populations increased according to flow cytometry analysis, indicating that the increased ALB expression after SCFAs treatment was not due to the selection of any specific cell population but rather the maturation of each cell.

Troglitazone was withdrawn from the market due to severe hepatotoxicity in humans, but this toxicity was not confirmed in previous nonclinical studies. CYP3A4-mediated severe hepatotoxicity related to troglitazone is well documented, and Tolosa et al. demonstrated a dose-dependent increase in troglitazone cytotoxicity following adenoviral transduction of CYP3A4 [40]. In the present study, our results showed that the promotion of metabolic activation by SCFAs treatment accurately revealed troglitazone toxicity in two different iPSC-derived liver organoids (Figure 4e and Figure S6c) and PHHs (Figure S7c). However, the phenomenon was not confirmed in HepG2 cells, which showed no expression of *CYP3A4* (Figure S7f). HepG2 cells tend to be more sensitive to several drugs but show poor accuracy for hepatotoxicity prediction, especially those occurring due to reactive metabolites generated by CYP enzymes [41]. PHHs exhibit a high expression level of CYP3A4. However, toxicity experiments using PHHs could not be conducted over 72 h due to rapid declines in cell viability. Therefore, these findings suggest that liver organoids, which allow for the repeated assessment of long-term drug treatment, can be used as a more clinically relevant human liver model.

This is the first study demonstrating that SCFA mixture of acetate, propionate, and butyrate increase CYP3A4 and ALB expression in human iPSC-derived liver organoids, and that these liver organoids, matured further by treatment with microbial metabolites, enabled early and precise prediction of drug toxicity. Lithocholic acid has been shown to bind and activate upstream transcriptional regulators of CYP enzymes, such as PXR and the farnesoid X receptor, directly [42]. Thus, further analysis of the precise molecular mechanism of the SCFAs-mediated gene expression changes should be performed. Investigation of liver organoids that can produce reactive toxic metabolites is ongoing. Moreover, we are attempting to develop niche-contained liver organoids, including sinusoidal endothelial cells, Kupffer cells, and hepatic stellate cells, to recapitulate and mechanistically analyze complex DILI responses. In the present form, our liver organoids may contribute to toxicity evaluation in personalized medicine and a better understanding of liver development and disease modeling.

## Data Availability

The data presented in this study are available in https://doi.org/10.3390/cells10010126.

## Supplementary Materials

The following are available online at https://www.mdpi.com/2073-4409/10/1/126/s1, Figure S1: Effects of acetate, propionate, and butyrate at various concentrations on *CYP3A4* and *CYP3A7* expression in iPSCs-derived liver organoids, Figure S2: Effects of acetate, propionate, and butyrate at various concentrations on *CYP* expression in iPSC-derived liver organoids, Figure S3: Effects of SCFA mixture on CMC-hiPSC-003-derived liver organoids, Figure S4: Effects of SCFA mixture on ALB expression and cell viability, Figure S5: Schedule of the optimization of liver organoid differentiation, Figure S6: Effects of SCFAs on the cell viability of CRL-2097-derived liver organoids and CYP3A4-mediated drug toxicity in CMC-hiPSC-003-derived liver organoids, Figure S7: Effects of SCFA mixture on PHHs and HepG2 cells, Table S1: Primer sequences used in this study, Table S2: Antibodies used in this study.
