# Supplementary material for: Effect of Microbial Short-Chain Fatty Acids on CYP3A4-Mediated Metabolic Activation of Human Pluripotent Stem Cell-Derived Liver Organoids
Source: Cells. 2021 Jan 11;10(1):126. doi: 10.3390/cells10010126 (PMC7827634; doi:10.3390/cells10010126)
Supplement: Supplementary file 1 [file cells-10-00126-s001.pdf]

Supplementary Figures

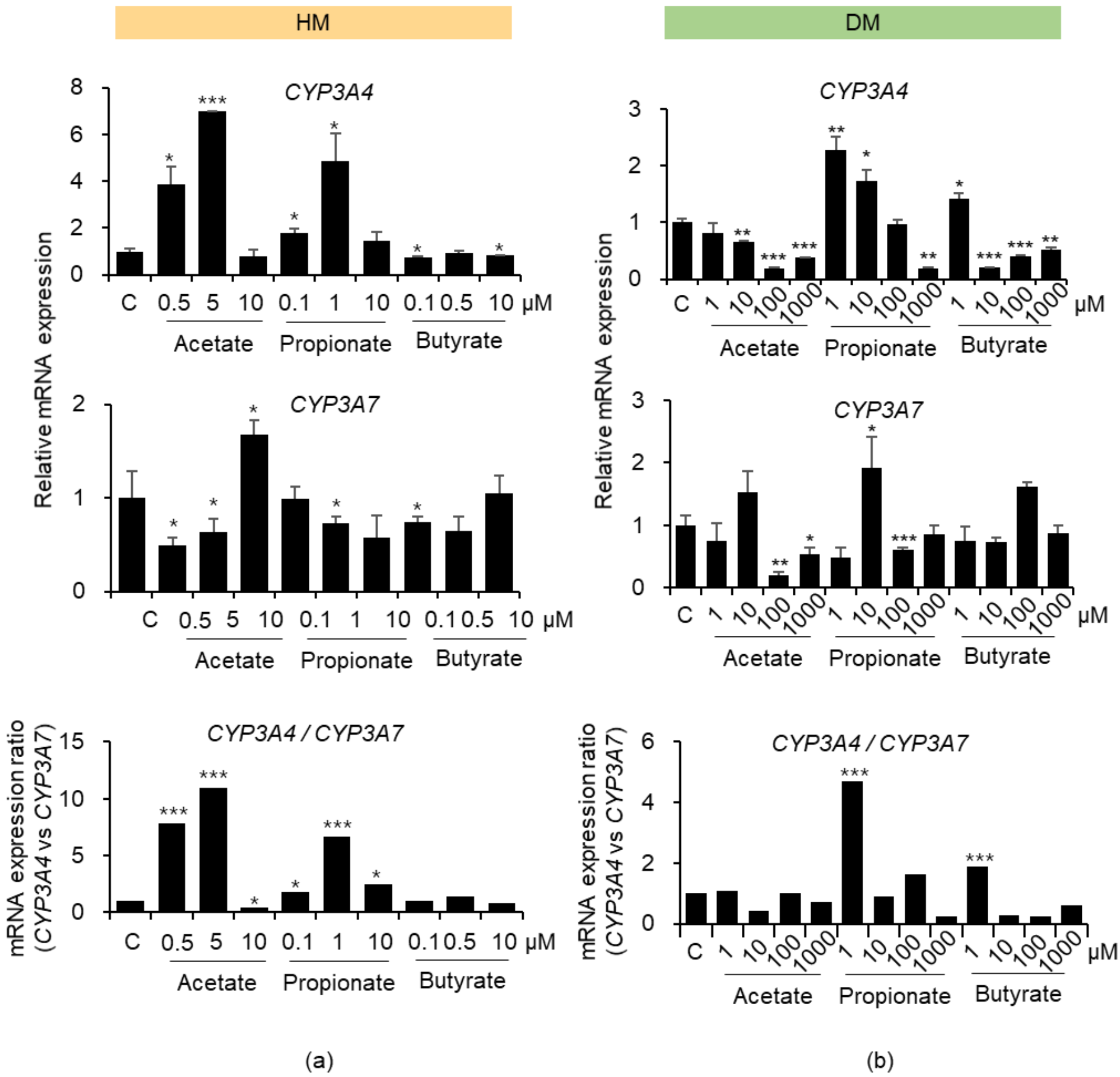

Figure 1. Effects of acetate, propionate, and butyrate at various concentrations on *CYP3A4* and *CYP3A7* expression in iPSCs-derived liver organoids. (**a**) mRNA expression levels of *CYP3A4* (*top*), *CYP3A7* (*middle*), and *CYP3A4*/*CYP3A7* ratio (*bottom*) in SCFAs-treated HM-cultured organoids at each indicated concentration. (**b**) mRNA expression levels of *CYP3A4* (*top*), *CYP3A7* (*middle*), and *CYP3A4*/*CYP3A7* ratio (*bottom*) in SCFAs-treated DM-cultured organoids at each indicated concentration. Data are presented as the mean ± SEM (n = 3) and analyzed by Student's t test. *$p < 0.05$, **$p < 0.01$, and ***$p < 0.001$.

.

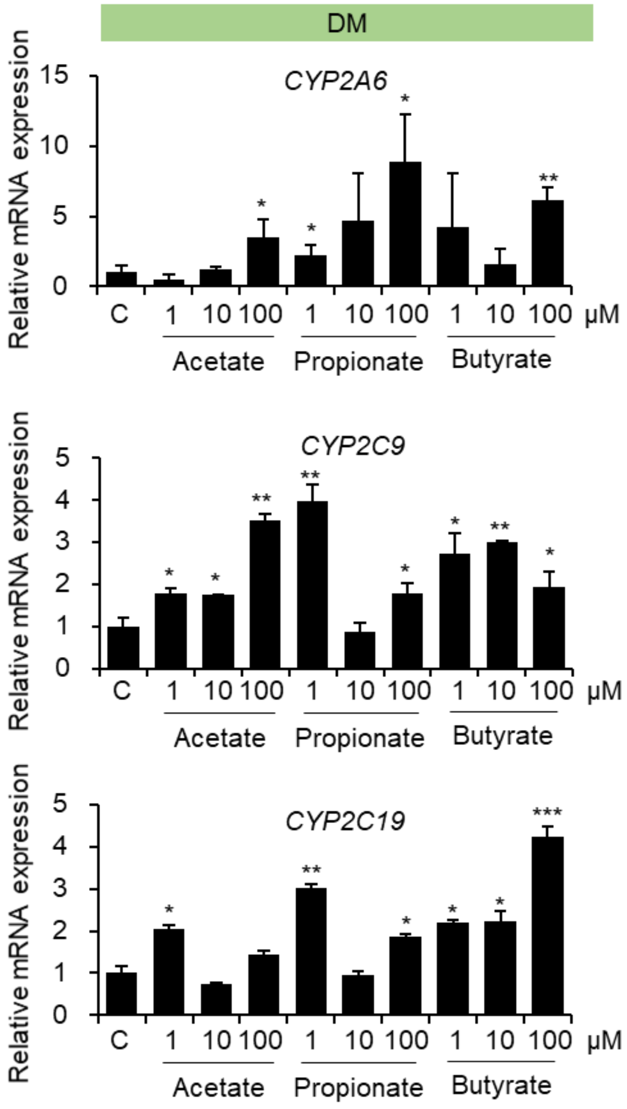

Figure S2. Effects of acetate, propionate, and butyrate at various concentrations on *CYPs* expression in iPSC-derived liver organoids. *CYP2A6* (*top*), *CYP2C9* (*middle*), and *CYP2C19* (*bottom*) mRNA expression levels in SCFAs-treated DM-cultured organoids at each indicated concentration. Data are presented as the mean ± SEM ($n = 3$) and analyzed by Student's t test. *$p < 0.05$, **$p < 0.01$, and ***$p < 0.001$.

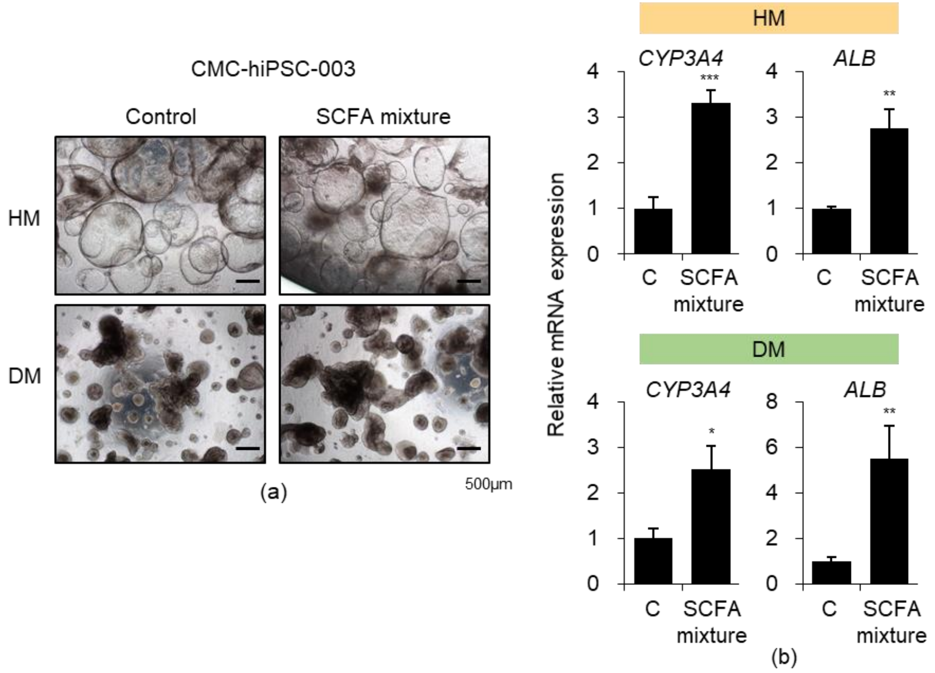

Figure S3. Effects of SCFA mixture on CMC-hiPSC-003-derived liver organoids. (**a**) Representative morphology of untreated control and SCFA mixture-treated HM (*upper*)- and DM (*lower*)-cultured organoids. (**b**) *CYP3A4* and *ALB* mRNA expression levels in control and SCFA mixture-treated HM (*upper*)- and DM (*lower*)-cultured organoids. Data are presented as the mean ± SEM ($n = 3$) and analyzed by Student's t test. *$p < 0.05$, **$p < 0.01$, and ***$p < 0.001$.

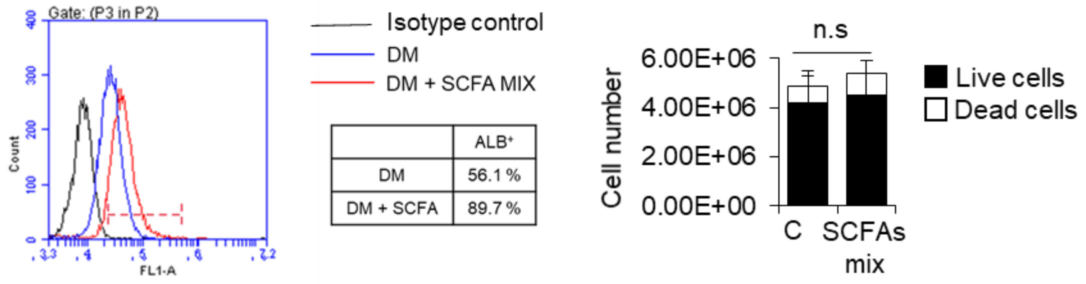

(a) (b)

Figure S4. Effects of SCFA mixture on ALB expression and cell viability. (**a**) Representative flow cytometry analysis of ALB-stained organoids in DM control and SCFA mixture treated groups. (**b**) Live/Dead cells in DM control and SCFA mixture treated organoids were counted after Trypan Blue staining.

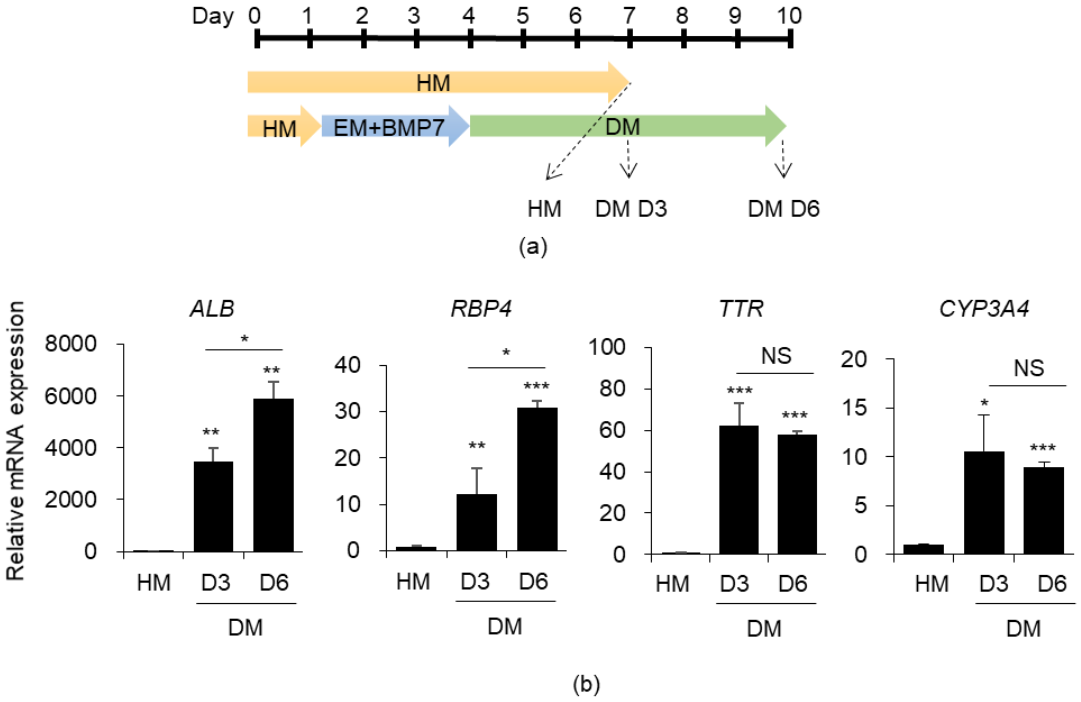

Figure S5. Schedule of the optimization of liver organoid differentiation. (**a**) Scheme of liver organoid differentiation. Hepatocyte marker-expression levels were examined at each indicated time point. (**b**) *ALB*, *RBP4*, *TTR*, and *CYP3A4* mRNA expression levels in HM-cultured organoids at D7, and DM-cultured organoids at D3 and D6 after differentiation. Data are presented as the mean ± SEM ($n = 3$) and analyzed by Student's t test. *$p < 0.05$, **$p < 0.01$, and ***$p < 0.001$.

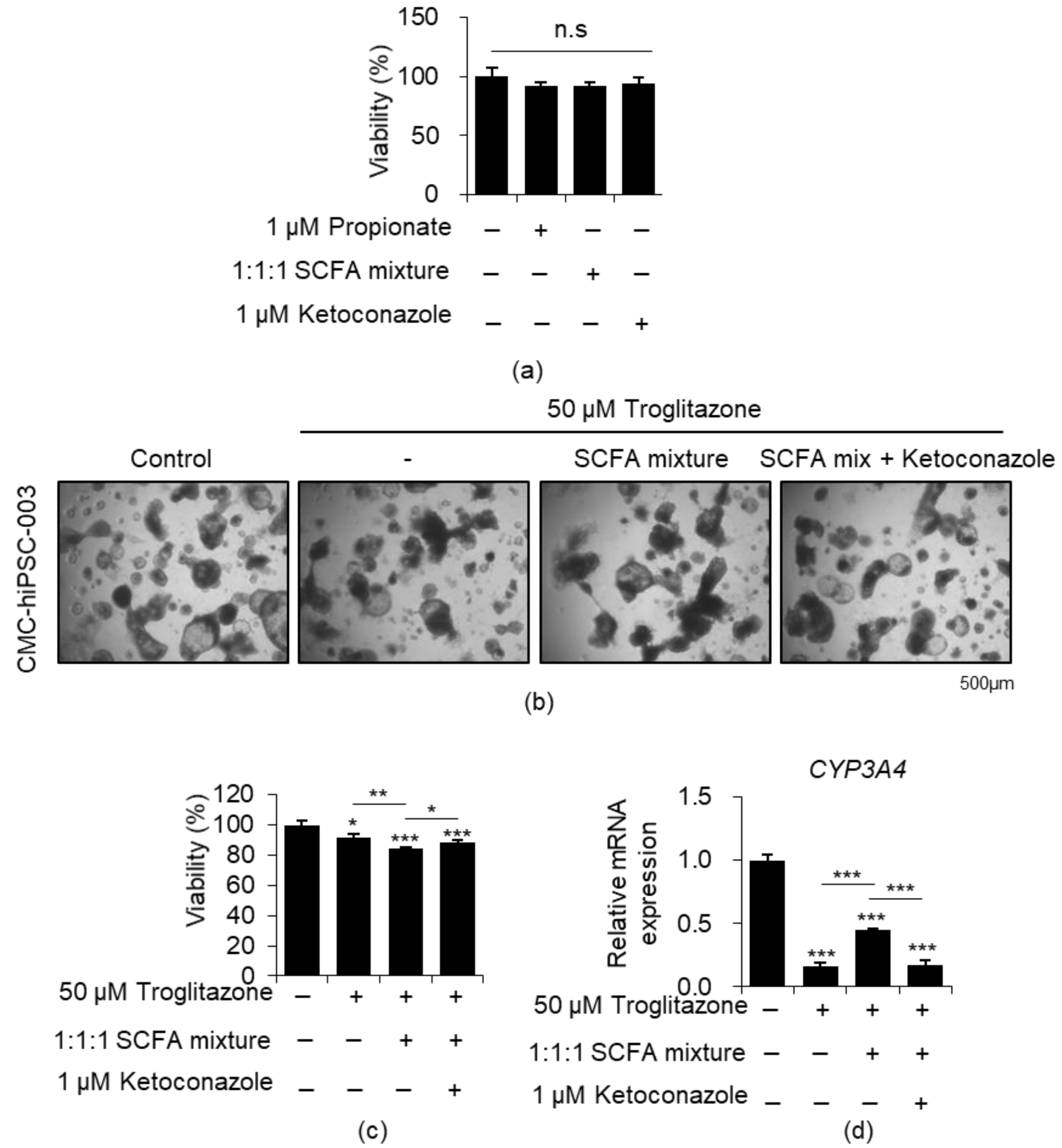

Figure S6. Effects of SCFAs on cell viability of CRL-2097-derived liver organoids and CYP3A4-mediated drug toxicity in CMC-hiPSC-003-derived liver organoids. (**a**) Cell viability was determined by cell counting in each treated DM-cultured organoids. (**b**) Representative morphology of DM-cultured CMC-hiPSC-003-derived liver organoids under each indicated treatment condition. (**c**) Relative cell viability under each indicated condition. (**d**) *CYP3A4* mRNA expression levels under each indicated condition. Data are presented as the mean ± SEM (n = 3) and analyzed by Student's t test. *$p < 0.05$, **$p < 0.01$, and ***$p < 0.001$.

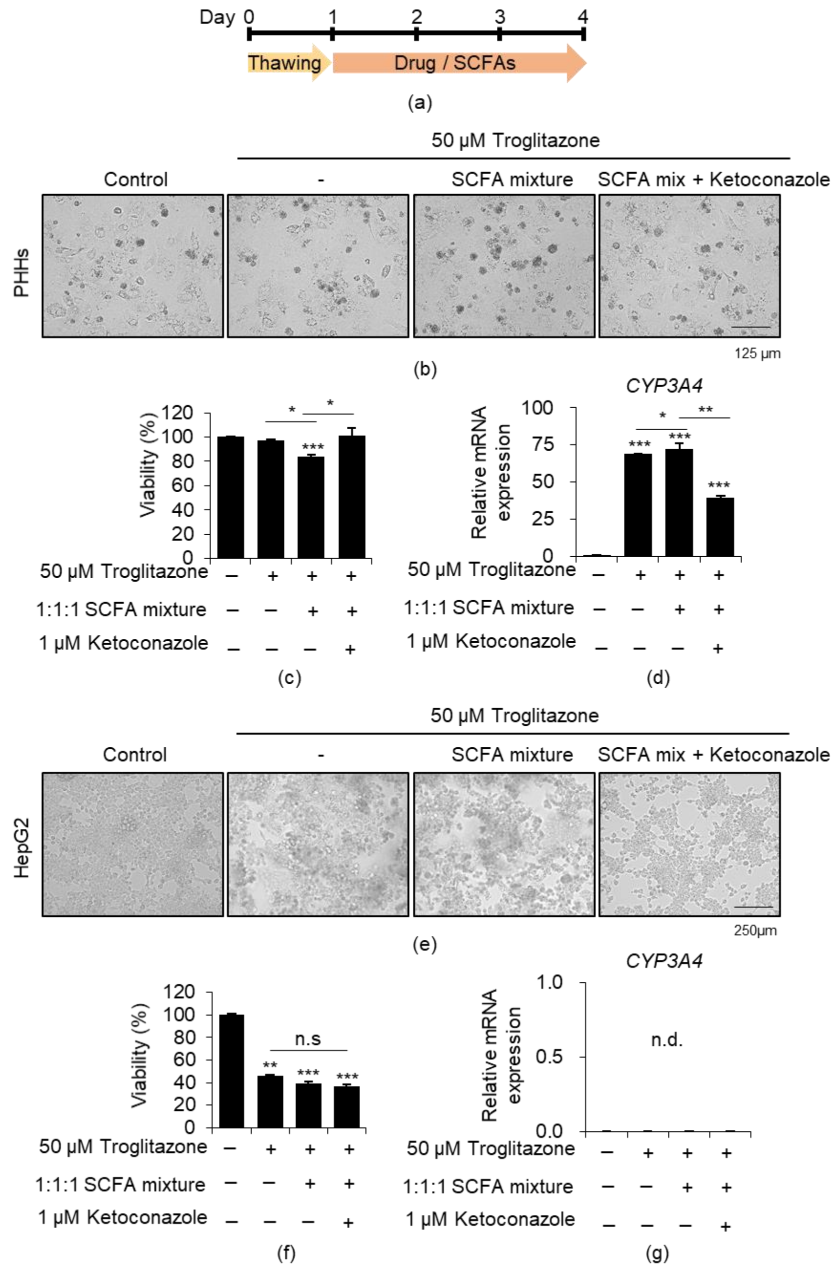

Figure S7. Effects of SCFA mixture on PHHs and HepG2 cells. (**a**) Scheme of drug and SCFAs treatment. (**b**) Representative morphology of PHHs under each indicated treatment condition. (**c**) Relative cell viability under each indicated condition. (**d**) *CYP3A4* mRNA expression levels under each indicated condition. (**e**) Representative morphology of HepG2 cells under each indicated treatment condition. (**f**) Relative cell viability under each indicated condition. (**g**) *CYP3A4* mRNA expression levels under each indicated condition. Data are presented as the mean ± SEM (n = 3) and analyzed by Student's t test. *$p < 0.05$, **$p < 0.01$, and ***$p < 0.001$.

Table S1. Primer sequences used in this study

Gene	Primer (Forward)	Primer (Reverse)
AFP	AGCTTGGTGGTGGATGAAAC	CCCTCTTCAGCAAAGCAGAC
ALB	TTTATGCCCCGGAACTCCTTT	AGTCTCTGTTTGGCAGACGAA
β-ACTIN	GGACTTCGAGCAAGAGATGG	AGCACTGTGTTGGCGTACAG
CK19	CGCGGCGTATCCGTGTCCTC	AGCCTGTTCCGTCTCAAACTTGGT
CYP2A6	CAGCACTTCCTGAATGAG	AGGTGACTGGGAGGACTTGAGGC
CYP2C9	CTACAGATAGGTATTAAGGACA	GCTTCATATCCATGCAGCACCAC
CYP2C19	ACAAGGGCAATCTGACTGGA	AGTGTTTCAGGTGGCTGGTA
CYP2D6	TGAAGGATGAGGCCGTCTGGGAGA	CAGTGGGCACCGAGAAGCTGAAGT
CYP3A4	CTTCATCCAATGGACTGCATAAAT	TCCCAAGTATAACACTCTACACAGACAA
CYP3A7	AAACTTGGCCGTGGAAACCT	CAGCATAGGCTGTTGACAGTC
HNF4A	GGCCAAGTACATCCCAGCTTT	CAGCACCAGCTCGTCAAGG
RBP4	GAGTTCTCCGTGGACGAGAC	TCCAGTGGTCATCATTTCCTTTC
TTR	TGGGAGCCATTTGCCTCTG	AGCCGTGGTGGAATAGGAGTA

Table S2. Antibodies used in this study

Antibodies	Company	Catalog No.	Dilution
anti-ALB	Bethyl Laboratories	A80-129a	1:100
anti-E-cadherin	BD Biosciences	610181	1:200
anti-HNF4A	Cell Signaling Technology	3113s	1:200
Anti-Ki67	Abcam	ab15580	1:200
